# Quasi-All-Passive Thermal Control System Design and On-Orbit Validation of Luojia 1-01 Satellite

**DOI:** 10.3390/s19040827

**Published:** 2019-02-17

**Authors:** Lin Yang, Qiang Li, Lin Kong, Song Gu, Lei Zhang

**Affiliations:** 1 Changchun Institute of Optics, Fine Mechanics and Physics, Chinese Academy of Sciences, Changchun 130033, China; yanglincas@163.com (L.Y.); konglin@charmingglobe.com (L.K.); gusong@charmingglobe.com (S.G.); 2 University of Chinese Academy of Sciences, Beijing 100039, China; 3 Chang Guang Satellite Technology Co., Ltd, Changchun 130102, China; li_qiang119@163.com

**Keywords:** quasi-all-passive, thermal analysis, thermal balanced test, on-orbit performance, temperature fluctuation

## Abstract

In order to resolve the large fluctuations in temperature range problem of Luojia 1-01 satellite caused by low heat inertia and poor thermal conductivity of structure, a quasi-all-passive thermal control system (TCS) design is presented under the conditions of limited resources including mass and power consumption. The effectiveness of the TCS design is verified by both ground thermal balanced test and related telemetry data of on-orbit performance. Firstly, according to the structural features and working modes of the satellite, isothermal design was implemented and the effectiveness was verified by thermal analysis using finite element method. Secondly, based on the results of the thermal analysis, thermal design was optimized and verified by the thermal balanced test. Finally, the thermal design was proved to be effective by temperature data acquired from telemetry data of on-orbit performance, and the thermal analysis model was improved and updated based on the results of thermal balanced test and temperature data of on-orbit performance. The on-orbit data indicates that temperature of optical camera stables at about 12 °C, temperature of battery stables at 19 °C, temperature of instruments inside and outside the satellite cabin is ranging from 10 °C to 25 °C. Temperature fluctuation range of optical camera is less than 2 °C when it is not imaging. Temperature fluctuation range of instruments not facing the sun is less than 4 °C. The data suggests that the temperature level of the satellite meets general design requirements, and the quasi-all-passive TCS design of the satellite is practicable.

## 1. Introduction

With the advantages of fast development cycle, economic and flexible characteristics, small and micro satellites are attracting more attention from commercial companies, institutes and universities [1]. Satellite fleet or satellite constellation which incorporates dozens or hundreds of small and micro satellites helps to improve the revisit cycle, and in turn makes the fleet or constellation more practical and useful nowadays [2,3,4,5,6]. Nevertheless, owing to the features of being small in volume, lightweight and with low heat inertia, coupled with their complicated working modes and limited satellite battery storage, it’s a great challenge to achieve a well performing satellite TCS [7,8,9].

Satellite TCS design is a critical part of the satellite. It helps to control the heat exchange between the satellite and the outside environment in order to maintain the temperature level of all the subsystems and instruments of the satellite [10]. Due to the limited onboard battery storage, low heat inertia and various work modes of Luojia 1-01 satellite, the mass and power allocated to the TCS is inadequate. As a result, it is unattainable to have as much active thermal control as it expects. It’s significant to present a quasi-all-passive TCS design of the satellite which characterized by light-weighted and low power consumption. A properly controlled temperature condition is critical for the satellite to perform its imaging tasks at nightlight time and daytime on-orbit.

This paper aims to present a quasi-all-passive TCS design and to verify the effectiveness of the design by ground thermal balanced test and on-orbit performance which can be gathered from the telemetry data according to the characteristics of Luojia 1-01 satellite. In the end the thermal model was improved and updated referring to the data of thermal balanced test and on orbit data. Normally, the subsystem design of satellites is always about getting more resources like mass and power consumption to have excessive safety margins, but sometimes this might end up causing redundancy and lead to an increase in cost. Compared with traditional TCS proposals, this paper comes up with a low-cost, low-energy thermal control system named quasi-all-passive TCS by adopting an isothermal cabin design method. The experiments and in-orbit data indicate is has good performance and effectiveness. In the end, this quasi-all-passive TCS provides an instance of reference for other thermal design of small and micro satellites which have limited mass and power for thermal control. 

## 2. TCS Design and Requirements

### 2.1. Definition of Satellite Coordinate System

**Figure 1:**
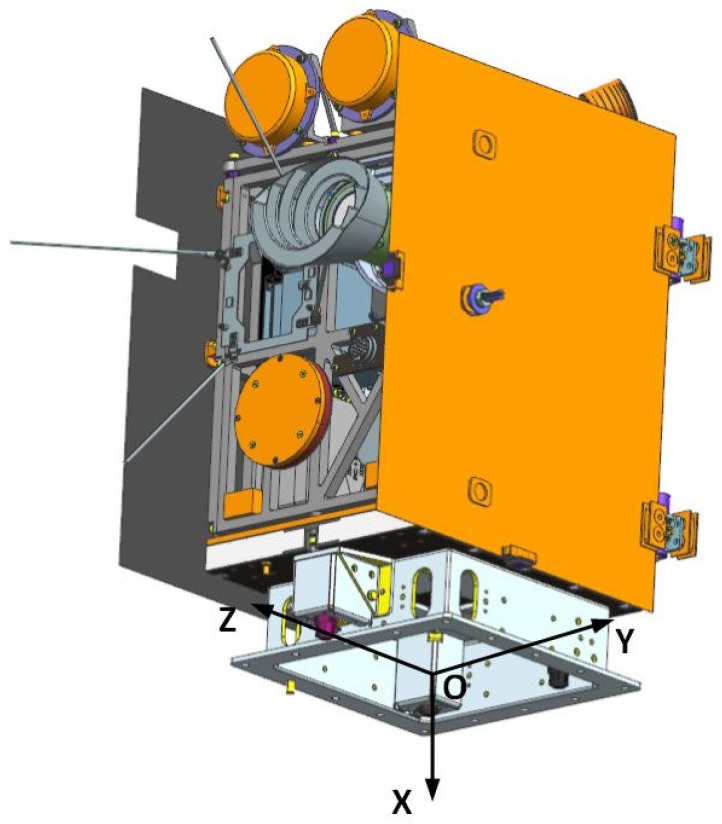
Luojia 1-01 satellite coordinate system.

**Figure 2:**
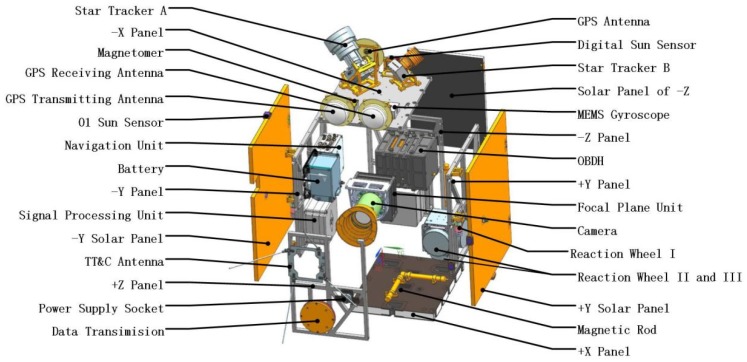
Exploded view of the Luojia 1-01 Satellite.

The whole satellite structure system adopts a frame structure design using lightweight aluminum alloy and weighs about 22 kg. The in-orbit status dimensions are 600 mm × 920 mm × 450 mm and the coordinate system is shown in Figure 1. The origin of the satellite coordinate system O is located in the geometric center of the bottom surface of the +X panel. Axis OZ lies in the bottom surface of the +X panel and it’s parallel to the optical axis of the satellite camera. Axis OX is vertical to the bottom surface of the +X panel and axis OY is determined by the Cartesian right-handed coordinate system. The OZY surface overlaps with the docking surface of the satellite and the launch vehicle and the explosive view of the satellite components is shown in Figure 2.

It’s clearly seen that the camera is mounted on the −X panel via a camera holder. The separation device is connected to the +X panel. There are two solar panels on the ±Y direction which are stowed on the surface of the satellite frame during the launch phase and shall be deployed by 90° when the satellite is in-orbit. There is a third solar panel stowed to the surface of the satellite frame in the direction of −Z. Inside the cabin of the satellite, there are on-board data handling system (OBDH), navigation system, battery, reaction wheel I, reaction wheel II and III, etc. Outside the cabin of the satellite, there are star tracker A, star tracker B, MEMS gyroscope, magnetometer, digital sun sensor, etc.

### 2.2. Relevant Thermal Parameters of TCS

The relevant thermal parameters of TCS, including type of orbit, satellite attitude during orbit, type of coatings and thermo-optical properties, element and thermal conductivity materials, thermal contact conductance coefficient between elements, equivalent thermal conductivity of multilayer heat insulation assembly (MLI), heat conductivity grease and heat conductivity membrane, etc. are listed in Table 1, Table 2, Table 3, Table 4 and Table 5.

### 2.3. Analysis of TCS Design Requirements

**Figure 3:**
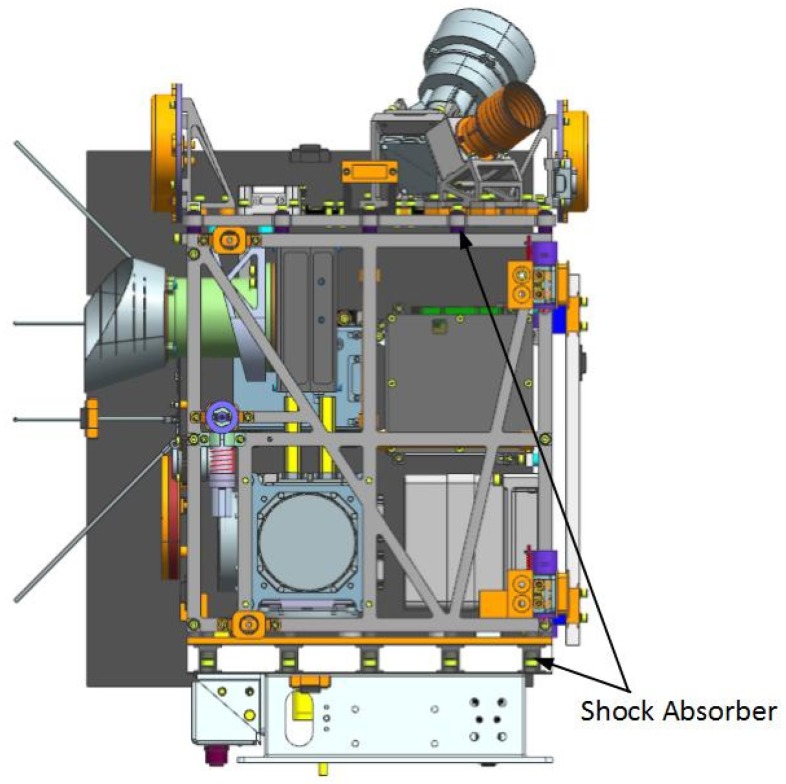
Shock absorbers in the Luojia 1-01 satellite (without the side solar panels).

The Luojia 1-01 satellite is a small satellite characterized by small volume, compact layout, a total mass is up to 22 kg, and the mass of the TCS is less than 1kg. It works in a Sun synchronous orbit (SSO) and its thermal stability is poor, which in turn makes the satellite more vulnerable to the space thermal environment. In the meantime, due to the lightweight design of the satellite structure, the contact surface between the instruments and the satellite structure is quite small. Moreover, as shown in Figure 3, there are some shock absorbers made of low thermal conductivity material, majorly butyl rubber, between the ±X panels and ±Y and ±Z panels, instruments and the panels they are meant to be mounted upon. As a consequence, the thermal conductivity is dramatically decreased on average. Coupled with the frequent shifting working modes of onboard instruments owing to the multiple work modes of the satellite, as a result, the temperature range fluctuation of the satellite is quite large.

The general thermal design requirements of the Luojia 1-01 are listed in Table 6. From the table, it can be concluded that inside the cabin, the camera and battery have for critical temperature demands, since the mass and power allocated to the TCS design system is too inadequate to have as much active thermal control as it needs. Consequently, there a majority of onboard instruments cannot have active thermal control priority. This paper then presents a quasi-all-passive TCS by adopting the isothermal cabin design method. That means the camera and the battery are the only ones who can have active thermal control, the rest of the onboard instruments are exposed to all-passive thermal control. It’s worth mentioning that no efficient heat transfer components like heat pipe can be used because of the satellite layout, mass and volume requirements.

In a word, the Luojia 1-01 satellite is a small satellite characterized by low heat inertia, large temperature fluctuation, poor thermal conductivity, bad instrument heat dispersion and limited resources, including mass and power allocated for TCS. Owing to these characteristics, a quasi-all-passive TCS design is presented and implemented. Doing this helps make sure all onboard instruments can function well by meeting all temperature requirements.

### 2.4. Analysis of the External Thermal Loads

**Figure 4:**
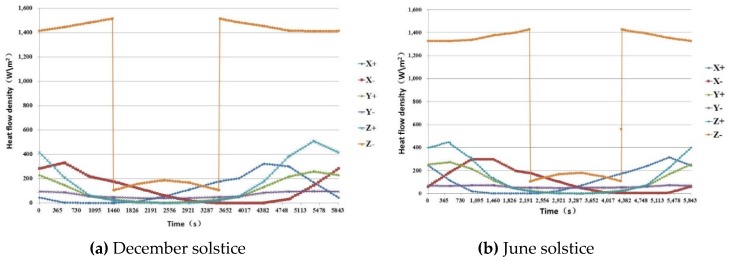
Variation curve of heat flux density on the satellite’s surface.

Given the structure, orbit parameters and flight attitude, the external thermal loads on the satellite’s external surface in long-term mode, the external thermal loads are calculated as orbital average heat flux density for all the surfaces, including direct solar radiation, albedo of the Earth and infrared radiation of Earth, as shown in Table 7, the variation curve of heat flux density is shown in Figure 4, parameters used in calculation as follows:

- Solar constant: $S=1412\;W/m^{2}\;\left(\mathrm{December}\;\mathrm{solstice}\right),S=1322\;W/m^{2}\left(\mathrm{June}\;\mathrm{solstice}\right)$;
- Earth infrared radiation: $E_{e}=0.25\left(1-\rho \right)S,\;\;\rho =0.30$;
- Earth albedo: $E_{r}=\rho S,\;\;\rho =0.30$;
- Earth radius: 6378.14 km;
- Space temperature: 4 K.

## 3. TCS Design Proposal

According to the previous analysis, taking all features including structure characteristics, thermal control requirements and heat flux of SSO into consideration, a technical proposal for the TCS was formed. Apart from the camera and battery, the rest of the satellite instruments are subject to all-passive thermal control. This proposal incorporates heat insulation design, heat conduction design, heat dispersion design and heating design. The general description of the technical proposal is stated below:

- The battery is mounted to the panel in a heat insulation way adopting an active heating circuit. Apart from the mounting surface to the panel, all surfaces are covered with MLI.
- OBDH is mounted to the panel via the thermal conductivity of the membrane.
- The brackets of star trackers are mounted to the panel in a heat insulation way. Brackets and star trackers are covered with MLI.
- The camera is mounted to the satellite structure through the camera holder in a heat insulation way. The active thermal control circuit and MLI are implemented on it.
- The baffle of the camera is covered with MLI and it’s connected to the camera in a heat insulation way.
- The focal plane unit is connected to the camera in a heat insulation way.
- Reaction wheels are mounted to the bracket in thermal conductive way using conductive grease in between the contact surfaces. The brackets are mounted on the panel in a heat insulation way.
- The navigation unit is mounted on the panel with thermal conductive grease in between the contact surfaces.
- The internal surface of the +X panel is covered with MLI. The external surface of the +X panel is covered with a thermal control coating named S781.

### 3.1. Heat Insulation Design 

Heat insulation design is mainly implemented by two ways, one is to cover instruments with MLI made of polyester film, and the other one is to insert heat insulating pads between contact surfaces of instruments and mounting panels. The external surface of the satellite frame is covered with MLI and the solar panels are mounted to the satellite frame via heat insulated pads. For instruments vulnerable to temperature change like the camera and battery, a heat insulation method is introduced in order to undermine or to isolate them from the influence of other instruments. Instruments outside the satellite cabin also employ the two ways of heat insulation with the purpose of diminishing the influence on them from the by outside space thermal environment which is also beneficial to keep the satellite isothermal. The regions and instruments covered by MLI are listed in Table 8 and the instrument mounting surfaces using heat insulated pads are listed in Table 9. 

### 3.2. Heat Conduction Design 

Due to the poor heat conduction status of the satellite panels and instruments, an isothermal design for all internal instrument of the satellite is presented given the characteristics of the layout inside the satellite cabin. To improve the equivalent thermal conductivity and thermal conductive membrane structure strength, conductive grease and multilayer conductive membranes are applied to the TCS. Regions and the method of conductivity and are listed in Table 10.

### 3.3. Heat Dispersion Design 

**Figure 5:**
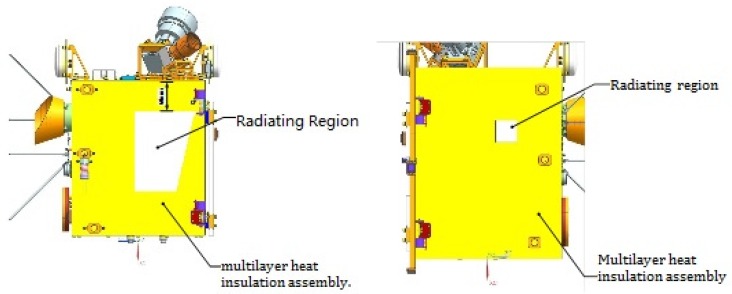
Radiating regions of the Luojia 1-01 satellite. (**a**) Radiating region of the +Y panel. (**b**) Radiating region of the −Y panel.

According to the typical heat flux of SSO and the location of the satellite separation device, the radiating surface is designated at the +Y panel near the OBDH location and −Y panel near the battery location, which are shown in Figure 5. The radiating region of +Y panel is 16,000 mm^2^ in trapezoid and the radiating region of the −Y panel is a 40 mm × 40 mm square. The coating of the internal surface of the radiating region is a heat conductivity membrane named HA95 and the external surface is a thermal control coating named F46.

### 3.4. Heating Design 

Since limited mass and power can be allocated to the TCS, there are only two heating regions for the whole satellite, as shown in Table 11. One is on the camera and the other is on the battery, both with backup for the heating instruments. The heater band is made of Constantan foil and the temperature measurement device is a thermistor.

## 4. Thermal Analysis

### 4.1. Finite Element Modeling

**Figure 6:**
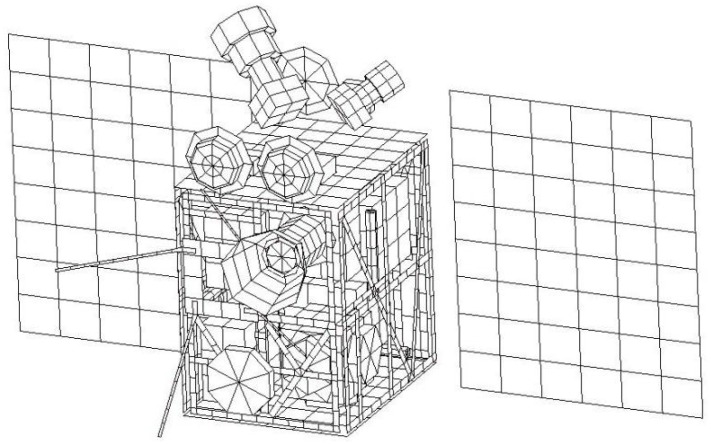
Thermal analysis model of the Luojia 1-01 satellite.

The finite element method is widely used in thermal analysis [11]. The finite element model is constructed in I-DEAS/TMG software shown in Figure 6. Since most of the satellite structures are in the form of a thin plate, the finite element model is majorly comprised of shell elements which can accelerate the computational efficiency. There are 3200 elements in total and the thickness of the elements is determined by equivalent structure thickness. In the process of finite element modeling, thermal coupling method is introduced in order to simplify the calculation and there are 55 thermal coupling in total.

### 4.2. Parameters of Working Condition in Thermal Analysis

First, the high and low temperature conditions are defined by the peaks and valleys of the solar constant in a year and the life expectancy of coatings [12]. The high temperature condition is defined like this, owing to the degeneration of the coatings at the end of the life expectancy, coupled with the peak of the solar constant at December solstice, the radiation regions absorbing the external heat flux is at the highest level. In the meantime, all the instruments are working at their maximum power consumption. The low temperature is defined like this. At the beginning of the life of the coatings, coupled with the valley of the solar constant at the June solstice, the radiation regions absorbing the external heat flux are at the lowest level. In the meantime, the satellite is in Sun pointing mode.

In the end, taking the long-term flight attitude, external heat flux and working modes of the satellite as a whole into consideration, the parameters and definition of temperature condition is defined in subsections 3.2.1 and 3.2.2.

#### 4.2.1. Condition I, High Temperature Condition

- Solar constant is at its peak, 1412 W/m^2^ (at December solstice)
- MLI made of polyimide, at the end of its life, αs/ε = 0.64/0.69 where αs means solar absorptance, ε means emissivity;
- At the end of the life of the radiation region coatings, αs/ε = 0.36/0.87;
- All instruments onboard are working at their maximum power consumption;
- Active thermal control is working well to meet the design requirement.

#### 4.2.2. Condition II, Low Temperature Condition

- Solar constant is at its valley, 1322 W/m^2^ (at June solstice)
- MLI made of polyimide, at the beginning of its life, αs/ε = 0.36/0.69;
- At the beginning of the life of the radiation region coatings, αs/ε = 0.17/0.87;
- All instruments onboard are working at their minimum power consumption;
- Active thermal control is working well to meet the design requirement.

### 4.3. Results of Thermal Analysis

**Figure 7:**
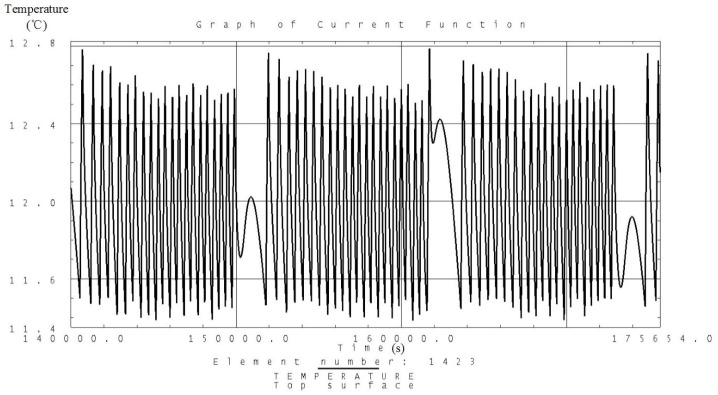
Analysis result of the temperature variation curve of the camera under high temperature conditions.

**Figure 8:**
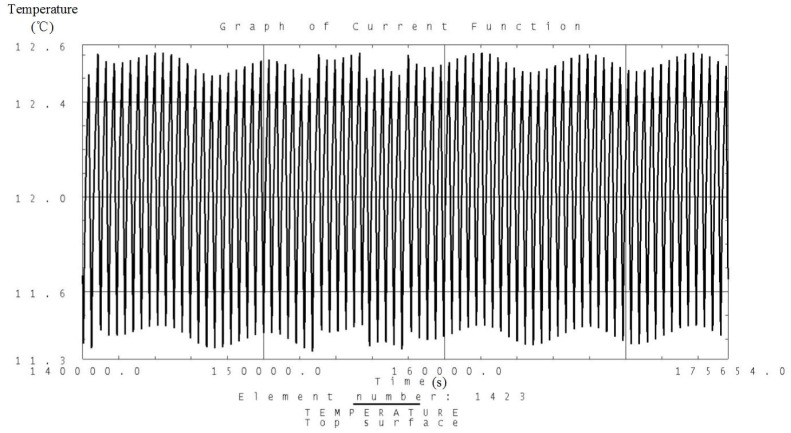
Analysis result of the temperature variation curve of the camera under low temperature conditions.

**Figure 9:**
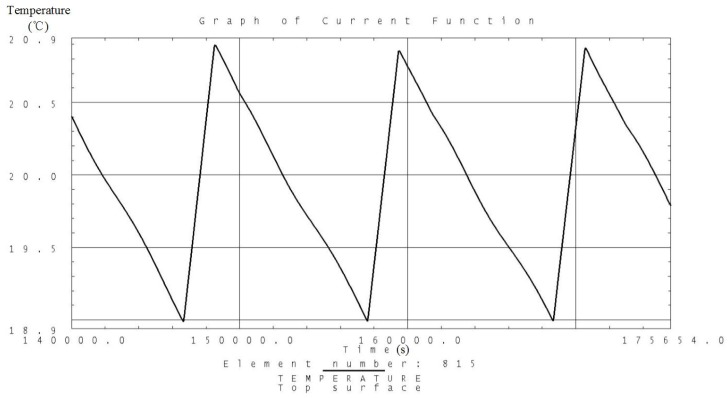
Analysis result of the temperature variation curve of the battery under high temperature conditions.

**Figure 10:**
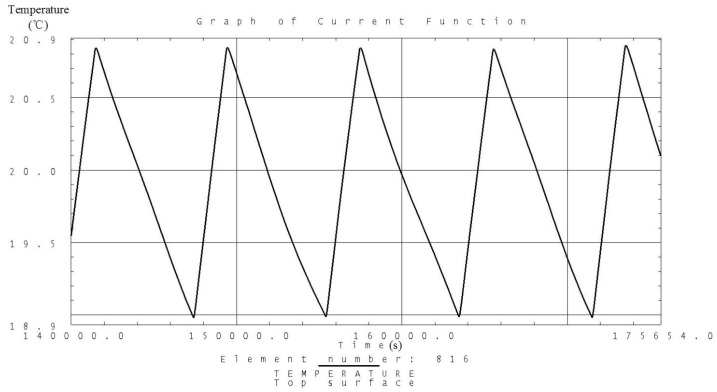
Analysis result of the temperature variation curve of the battery under low temperature conditions.

**Figure 11:**
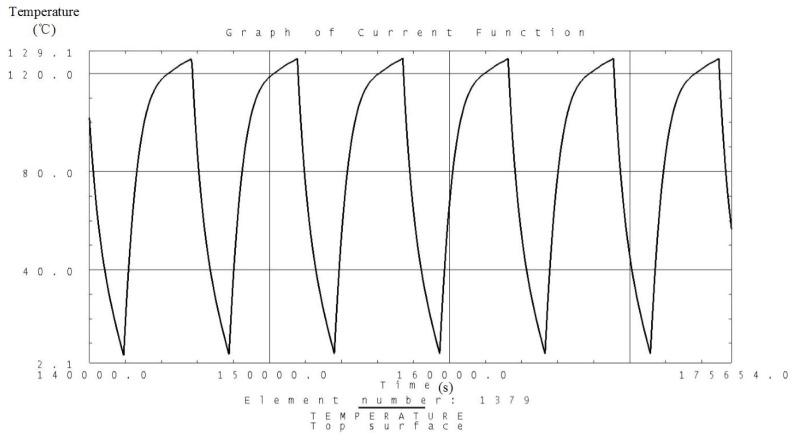
Analysis result of the temperature variation curve of the solar panels under high temperature conditions.

**Figure 12:**
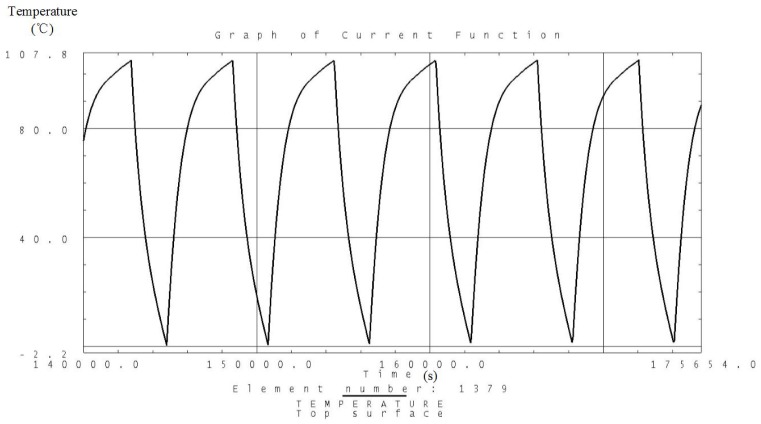
Analysis result of the temperature variation curve of the solar panels under low temperature conditions.

Analysis is carried out according to the thermal control measures and defined workloads including the high and low temperature conditions. Temperature variation curves of the camera, battery and solar panels are obtained and shown in Figure 7, Figure 8, Figure 9, Figure 10, Figure 11 and Figure 12 and temperature variation conditions of instruments inside and outside the satellite cabin is shown in Table 12.

From the above figures and tables, it can be concluded that during long term working shifts from high to low temperature conditions, every instrument is working under the required temperature by the present quasi-all-passive TCS even with some design margin. The temperature of the camera stables at 12 °C and battery stables at the range from 18 °C to 20 °C. Temperature of instruments inside the satellite cabin is ranging from 5 °C~30 °C and the fluctuation is better than 5 °C.

## 5. Experimental Verification

To validate the TCS design of the Luojia 1-01 satellite, and also to provide reference for model modification, the flight model of the satellite has gone through the ground thermal balanced test. Satellite on-orbit performance is normally predicted by thermal balanced test.

### 5.1. Test Devices and Site

**Figure 13:**
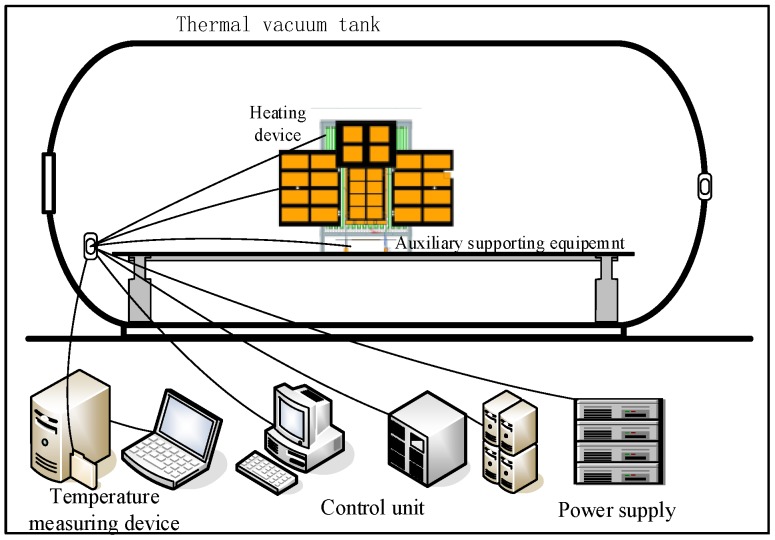
Schematic diagram of the testing devices.

**Figure 14:**
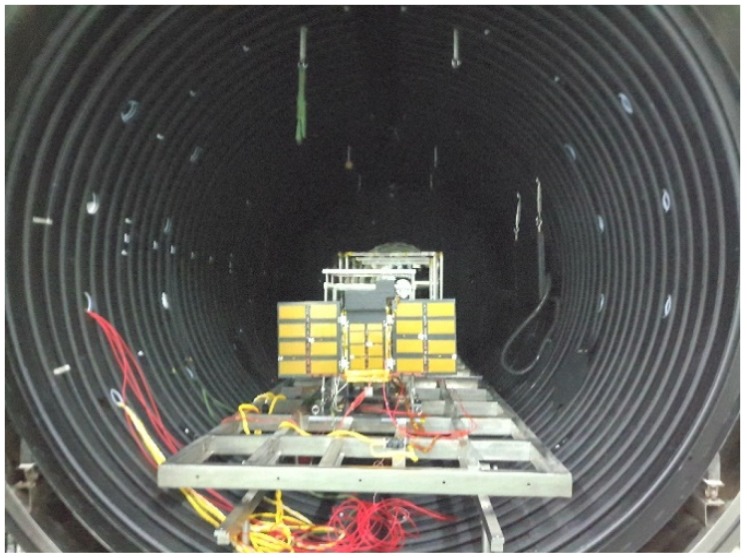
Testing site of Luojia 1-01.

In Figure 13, there are devices for thermal balanced test including flight model, heating devices, auxiliary equipment, temperature measuring device, power supply, control unit, thermal vacuum tank, etc. Test site is shown in Figure 14. The temperature of chamber is under 100 K, and the pressure is below 10^−5^ Pa. Heat flux of the sun is mainly performing on the solar panels, other surfaces in this direction is covered with MLI, influence of heat flux can be ignored. The effect of the sun is simulated by heating the solar panels to reach the temperature level on orbit.

### 5.2. Testing Working Conditions

High temperature conditions in the thermal balance test simulate the external heat flux during flight on the December solstice, under the condition that all the instruments are working at their maximum power consumption status and the properties of coatings are at the end of their life expectancy. The parameters of the high temperature conditions in the thermal balance test are set as follows:

- The camera continues to work for a duration of 120 s in the shadow region and the data transmission unit continues to work for a duration of 300 s in the sunlight region. All the rest of the instruments keep on working at their maximum power consumption except the navigation unit which is working at the minimum power consumption
- Active thermal control is working as designed. The temperature of the camera remains stable at 12 °C and the battery temperature is stable at 18 °C.
- The external heat flux is set the same as the high temperature condition in the thermal analysis using FEM.
- The properties of the coatings and MLI is set up as the ones at the end of their life expectancy.

Low temperature conditions in the thermal balanced test simulate the external heat flux during flight on the June solstice, under the condition that all the instruments are working at their minimum power consumption status and the coatings are at a good status in the beginning of its life expectancy. The low temperature condition parameters in the thermal balance test are set as follows:

- The camera is off and the data transmission is off. All the rest of the instruments keep on working at their minimum power consumption.
- Active thermal control is working as designed. The temperature of the camera is stable at 12 °C and the battery is stable at 18 °C.
- The external heat flux is set the same as the low temperature conditions in the thermal analysis using FEM.
- The properties of the coatings and MLI is set up as the ones at the beginning of their life expectancy.

### 5.3. Test Results

The temperatures of the instruments of the satellite are obtained during the thermal balance test in both high temperature conditions and low temperature conditions. There are two measuring points on both camera and battery which are considered to be critical instruments to the satellite system. It can be concluded from the Table 13 that all the temperatures of instruments are well controlled. The highest temperature, which is 29.3 °C, appears on the navigation unit during high temperature conditions and the lowest temperature, which is −0.5°C, appears on the star tracker A during low temperature conditions.

The duty cycle and power consumption of the heating regions of active heat control are worth paying attention to. The parameters are shown in Table 14. It can be seen that the power consumption is 1.28 W and 2.72 W, respectively, under high and low temperature conditions. Because of the extremeness of the high temperature conditions, the power consumption at low temperature is a little bit over the maximum satellite power consumption.

## 6. Analysis of in-Orbit Performance and Modeling Modification

The Luojia 1-01 satellite was launched into orbit on 2 June 2018 from the Jiuquan launch site, in China. Since it was deployed successfully, all systems have been operating smoothly. The temperatures of instruments inside and outside the satellite cabin are at a rational level. By analyzing the telemetry data related to temperature, and comparisons to the ground thermal balance test data, the thermal model is further modified and updated.

### 6.1. On-Orbit Data Analysis

**Figure 15:**
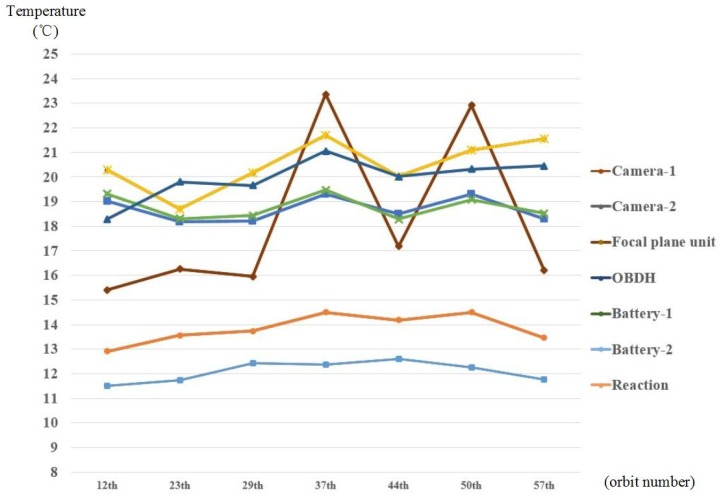
Temperature variation curve of the inside instruments on the Luojia 1-01.

The temperature variation curve of inside instruments of the satellite is shown in Figure 15. Among them, owing to the imaging tasks and data transmission tasks at the 37th orbit and 50th orbit, the temperature of the focal plane unit and OBDH is increasing apparently. The temperature of focal the plane unit is as high as 23.4 °C and the OBDH is at 22.7 °C. The temperatures of the camera and battery is stable under control and temperature fluctuation is better than 2 °C when the camera is not imaging.

**Figure 16:**
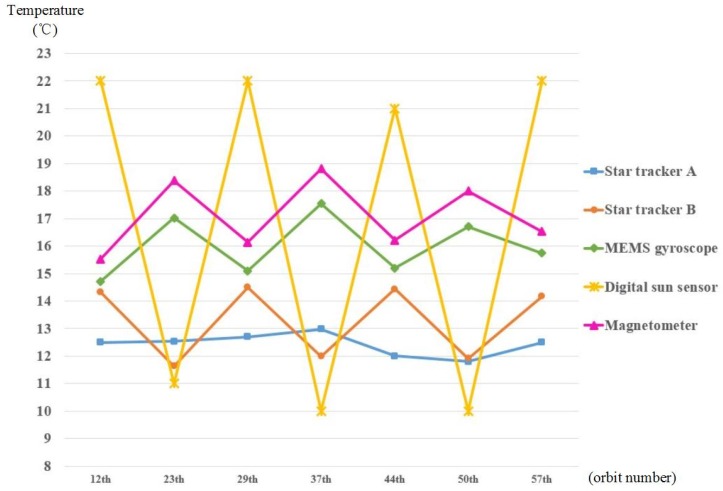
Temperature variation curve of the outside instruments in the Luojia 1-01.

The temperature variation curve of outside instruments of the satellite is shown in Figure 16. Star tracker A is stable in the range from 11.8 °C to 13.0 °C. Star tracker B is stable in the range from 11.6 °C to 14.5 °C. The MEMS gyroscope is stable in the range from 14.7 °C to 17.5 °C. The digital Sun sensor is stable in the range from 10.0 °C to 22.0 °C. The magnetometer is stable in the range from 15.5 °C to 18.4 °C. Temperature fluctuations are in line with expectations.

Taking all data from the in-orbit balanced status, ground thermal tests and thermal analysis into consideration, the model needs to be improved and updated. The comparison data is shown in Table 15.

It can be seen from Table 15 that the temperature of each measuring point in-orbit has a margin of less than 8 °C compared to the high temperature conditions in the test, and a margin of less than 15 °C compared to the low temperature conditions in the test. All temperatures of in-orbit instruments fall in between the high and low temperature condition results. It indicates the TCS design is valid and the results of the ground thermal balance tests and thermal analysis are correct and within tolerance.

The power consumption for active control and comparison to the ground thermal balance test and thermal analysis are shown in Table 16. The average power consumption is 1.68 W, which falls in between the high and low temperature conditions of the ground tests while not exceeding 2 W.

### 6.2. Thermal Model Modification 

Based on the in-orbit performance temperature data, the key thermal analysis parameters such as thermal coupling value, surface radiation properties, equivalent thermal conductivity of MLI and power consumption of instruments are amended and Monte Carlo methods and sensitivity analysis are introduced in this process [13]. 

First the parametric sensitivity analysis is completed by the Monte Carlo method. Then according to the thermal balance test and in-orbit data, the Latin hypercube sampling and simplex mixed methods are applied to correct the model parameters by the classification layer by layer, and the optimal values are calculated. The amended results and comparison before and after the changes is shown in Table 17. From the comparison, it can be seen that the difference between in-orbit data and the ground test and thermal analysis data is within 3 °C, which indicates the model modification is effective.

## 7. Conclusions

By adopting the presented quasi-all-passive TCS for the Luojia 1-01 satellite we have successfully addressed the problem of low heat inertia and high temperature fluctuation with very limited resources allocated for thermal control. As a result, the key instruments like the camera and battery can function well in a preferable temperature environment. Moreover, the successful application of flexible heat conducting material addressed the poor heat conduction between instruments and mounting panels because of the use of shock absorber devices.

In-orbit temperature data indicates that the camera is stable at 12 °C, the battery is stable at 19 °C, and the instruments outside the satellite cabin range from 10 °C to 25 °C. The inside temperature fluctuation of the satellite when not facing the Sun is stable at 4 °C when the camera is not working. Taking advantage of the in-orbit temperature data, the thermal model is further improved and the result after amendment shows that temperature difference is controlled within 3 °C. Finally, the in-orbit data is close to the data obtained from ground thermal balance test and thermal analysis. The quasi-all-passive TCS proves to be effective and has reference value for small and micro satellite TCS design.

## Figures and Tables

**Table 1:** Orbit parameters of satellite in general.

Subjects	Parameters
Orbit height	645 km
Orbit eccentricity	0
Orbit inclination	98.04˚
Orbit period	5855.15 sec
Local time of descending node	10:30 am

**Table 2:** Flight mode and attitude of the satellite orbit.

Flight Mode	Attitude	Working Mode
Sun pointing	-OZ axis points to the Sun, -OY axis points to the orbit normal, OX axis is defined by the right-hand rule	Long-term
Nadir pointing	-OZ axis points to the Earth, -OY axis points to the orbit normal, OX axis is defined by the right-hand rule	Short-term

**Table 3:** Material properties of the thermal subsystem.

Materials	Thermo-Optical Properties	Thermal Conductivity
Aluminum coated polyimide film (one side)	$\alpha_{s}=0.36,\varepsilon_{H}=0.69$	——
Argentum coated F46 film (one side)	$\alpha_{s}=0.12,\varepsilon_{H}=0.64$	——
2A12 (black coated)	$\varepsilon_{H}=0.85$	121 W·m^−1^K^−1^
2A12 (Natural aluminum anodized)	$\varepsilon_{H}=0.1$	121 W·m^−1^K^−1^
ZT4 (black coated)	$\varepsilon_{H}=0.85$	8.8 W·m^−1^K^−1^
T700 (black painted)	$\varepsilon_{H}=0.92$	5 W·m^−1^K^−1^

**Table 4:** Thermal contact conductance coefficient between elements.

Subjects	Contacting Form	Thermal Contact Conductance Coefficient
Camera and satellite platform	Point contact/rough interface	10 W/(m^2^·K)
Battery and satellite platform	Point contact/rough interface	10 W/(m^2^·K)
Antenna stand and satellite platform	Point contact/rough interface	10 W/(m^2^·K)
Structures and structures	Surface contact/smooth interface	100 W/(m^2^·K)
Instruments and platform	Surface contact/smooth interface/heat conductivity grease	1000 W/(m^2^·K)

**Table 5:** Equivalent thermal conductivity of MLI and heat conduction materials.

Subjects	Equivalent Thermal Conductivity
MLI (20 units)	0.04 W/(m·K)
MLI (10 units)	0.048 W/(m·K)
Heat conductivity grease	3 W/(m·K)
Heat conductivity membrane (horizontal direction)	1000 W/(m·K)

**Table 6:** Parameters of TCS design requirements in general.

Subjects	Parameters
Temperature inside the cabin	−10~+45 °C
Temperature of the battery	10~+30 °C
Temperature of star trackers	−30~+30 °C
Temperature of camera body	0~+30 °C
Temperature of solar panel	−85~+135 °C
Temperature of antennas	−90~+90 °C
Mass allocated to thermal control	≤1 kg
Power allocated to thermal control	≤2 W

**Table 7:** Average heat flux density (W/m^2^).

Surface	December Solstice				June Solstice			
	Sun	Albedo	Infrared	Sum	Sun	Albedo	Infrared	Sum
+X	0	37.8	76.2	114.0	0	34.5	76.1	110.6
−X	0	37.8	76.0	113.8	0	34.4	76.1	110.5
+Y	0	46.2	49.4	95.6	0	46.8	52.2	99.0
−Y	0	20.2	43.3	63.5	0	14.8	44.1	58.9
+Z	0	77.1	72.1	149.2	0	64.1	69.1	133.2
−Z	891.5	5.3	74.6	971.4	859.3	5.4	72.9	937.6

**Table 8:** Regions and instruments of Luojia 1-01 covered by MLI.

Regions and Instruments	Amount of Layers
−X panel, ±Y panel, ±Z panel	20
Internal surface of +X panel	20
Baffle of the camera	20
Camera and its bracket	20
Battery (except the mounting suface)	20
Star trackers and its brackets (2)	20
Brackets of the GPS Antennas (3)	20
Bracket of the digital sun sensor	20

**Table 9:** Description of instruments mounting surfaces using heat insulated pads.

Subject	Material of Heat Insulated Pads
Mounting surface between camera bracket and −X panel	Polyimide
Mounting surface between baffle and camera	Polyimide
Mounting surface between camera and focal plane unit	Titanium alloy
Mounting surface between battery and −Y panel	Polyimide
Mounting surface between brackets of star trackers (2) and −X panel	Polyimide
Mounting surface between GPS brackets (3) and −X panel	Polyimide
Mounting surface between digital Sun sensor bracket and −X panel	Polyimide
Mounting surface between data transmission antenna and +Z panel	Polyimide
Mounting surface between TT&C antenna and +Z panel	Polyimide
Mounting surface between side −Z solar panel and −Z panel	Polyimide

**Table 10:** Description of regions and method of thermal conductivity applied in Luojia 1-01.

Subject	Heat Conductivity Method
Mounting surface among ±Y and ±Z panels	Heat conductivity grease in contact surface and stickup of heat conductivity membrane
Mounting surface between OBDH and −Z panel	Heat conductivity grease in contact surface and stickup of heat conductivity membrane
Mounting surface between navigation unit and −Y panel	Heat conductivity grease in contact surface and stickup of heat conductivity membrane
Mounting surface between reaction wheels and +Y panel	Heat conductivity grease in contact surface and stickup of heat conductivity membrane
Mounting surface between MEMS gyroscope and −X panel	Heat conductivity grease in contact surface and stickup of heat conductivity membrane
Mounting surface between magnetometer and −X panel	Heat conductivity grease in contact surface and stickup of heat conductivity membrane
Mounting surface between star trackers and brackets	Stickup of heat conductivity membrane
Mounting surface between −Z panel and internal surface of the +X panel	Stickup of heat conductivity membrane

**Table 11:** Parameters of the heating regions of the Luojia 1-01 satellite.

No.	Subject	Location	Temperature Threshold (°C)	Nominal Power Consumption (W)
1	Heating region of the camera	Tube of the camera	11.5~12.5	2
2	Backup for the heating region of camera	Tube of the camera	9.5~10.5	2
3	Heating region of the battery	Shell of the battery	18.0~20.0	2
4	Backup for the heating region of the battery	Shell of the battery	15.0~17.0	2
			Sum	8

**Table 12:** Analysis result of the temperature variation conditions of instruments inside and outside the satellite cabin.

Location	Instrument	Condition I (°C)	Condition II (°C)	Required Temp (°C)
Inside the satellite	OBDH	19.1~23.4	10.2~15.3	−10~45
	Reaction wheel I	21.0~27.2	12.7~16.6	−10~45
	Reaction wheel II	14.0~17.5	8.2~12.1	−10~45
	Navigation unit	22.4~28.0	13.5~17.8	−10~45
	Signal processing unit	18.8~20.9	9.5~14.4	−10~45
Outside the satellite	MEMS gyroscope	16.8~18.3	10.2~11.3	−10~45
	Magnetometer	10.1~12.6	5.7~7.9	−10~45
	Star tracker A	14.8~16.2	6.7~9.2	−30~30
	Star tracker B	15.5~17.4	9.7~12.1	−30~30

**Table 13:** Temperatures of the onboard instruments in thermal balance test.

No.	Thermoscope	Subject	High Temp Condition (°C)	Low Temp Condition (°C)
1	T1 (Thermistor)	Camera-1	12.4	11.9
2	T2 (Thermistor)	Camera-2	13.9	10.8
3	T3 (Thermistor)	Focal plane unit	24.9	7.7
4	T4 (Thermistor)	OBDH	26.7	6.1
5	T5 (Thermistor)	Battery-1	19.9	18.2
6	T6 (Thermistor)	Battery-2	20.6	18.4
7	T7 (Thermistor)	Reaction wheel I	28.0	8.3
8	T8 (Thermistor)	Star tracker A	18.4	−0.5
9	T9 (Thermistor)	Star tracker B	20.3	0.3
10	TC1 (thermocouple)	Camera holder	13.5	9.0
11	TC2 (thermocouple)	Reaction wheel II	20.7	4.3
12	TC3 (thermocouple)	Navigation unit	29.3	5.5
13	TC4 (thermocouple)	Signal processing unit	21.4	5.2
14	TC5 (thermocouple)	Magnetometer	15.3	2.6
15	TC6 (thermocouple)	MEMS gyroscope	20.3	7.2

**Table 14:** The duty cycle and power consumption of the heating regions.

No.	Subject	Heating Circuit	Assigned Power (W)	Duty Cycle		Actual Power Consumption (W)	
				High Temp	Low Temp	High Temp	Low Temp
1	Camera-1	H1	2	0.34	0.70	0.68	1.4
2	Camera-2	H2	2	0	0	0	0
3	Battery-1	H5	2	0.30	0.66	0.60	1.32
4	Battery-2	H6	2	0	0	0	0
sum						1.28	2.72

**Table 15:** Temperature of instruments from on-orbit, ground thermal balanced test and thermal analysis.

Subject	On-orbit Balanced Temp (°C)	High Temp in Test (°C)	Low Temp in Test (°C)	High Temp in Thermal Analysis (°C)	Low Temp in Thermal Analysis (°C)
Camera-1	12.20	12.4	11.9	12.8	11.3
Camera-2	12.94	13.9	10.8	14.0	12.0
Focal plane unit	18.65	24.9	7.7	24.8	7.4
OBDH	20.54	26.7	6.1	23.4	10.2
Battery-1	18.63	19.9	18.2	20.7	18.6
Battery-2	18.68	20.6	18.4	21.2	18.0
Reaction wheel I	20.22	28.0	8.3	27.2	15.0
Star tracker A	12.72	18.4	-0.5	14.2	6.7
Star tracker B	13.11	20.3	0.3	17.4	9.7

**Table 16:** Temperature of instruments from on-orbit, ground thermal balanced test and thermal analysis.

Heating Region	Designed Power Consumption (W)	On-Orbit Power Consumption (W)	Power in High Temp of Test (W)	Power in Low Temp of Test (W)	Power in High Temp of Thermal Analysis (W)	Power in Low Temp of Thermal Analysis (W)	
Heating region of the camera	2	0.48	0.36	1.4	0.30	1.18
Backup heating region of the camera	2	0	0	0	0	0
Heating region of the battery	2	1.20	0.86	1.32	0.78	1.34
Backup heating region of the battery	2	0	0	0	0	0
sum	8	1.68	1.22	2.72	1.08	2.52

**Table 17:** Amendment comparison of temperature of instruments from in-orbit and thermal analysis.

Subject	On-orbit Balanced Temp (°C)	High Temp in Thermal Analysis (°C)		Low Temp in Thermal Analysis (°C)	
		Before	After	Before	After
Camera-1	12.20	12.8	12.5	11.3	11.9
Camera-2	12.94	14.0	13.2	12.0	12.5
Focal plane unit	18.65	24.8	20.8	7.4	15.9
OBDH	20.54	23.4	22.3	10.2	18.4
Battery-1	18.63	20.7	19.2	18.6	18.6
Battery-2	18.68	21.2	19.7	18.0	18.5
Reaction wheel I	20.22	27.2	23.1	15.0	17.9
Star tracker A	12.72	14.2	13.0	6.7	9.9
Star tracker B	13.11	17.4	15.5	9.7	11.4
